# Burning Mouth Syndrome: An Overview and Future Perspectives

**DOI:** 10.3390/ijerph20010682

**Published:** 2022-12-30

**Authors:** Daniela Adamo, Gianrico Spagnuolo

**Affiliations:** Department of Neurosciences, Reproductive and Odontostomatological Sciences, University of Naples Federico II, 80138 Napoli, Italy

Burning Mouth Syndrome (BMS) is a complex chronic neuropathic orofacial pain disorder characterized by a generalized or localized intraoral burning, dysesthetic sensation or pain of the oral mucosa, recurring daily for more than 2 h per day for more than 3 months, without any evidence of specific mucosal lesions and/or laboratory findings [1]. Various terminologies have been used to describe the burning sensation in the mouth, especially referring to the tongue, such as glossodynia, burning tongue, stomatodynia, oral dysaesthesia and complex oral sensitivity disorders [2,3]. The worldwide prevalence of BMS is 1.73% in the general population, almost 8% in clinical patients, affecting predominantly middle-aged and older women peri and postmenopausal with a female to male ratio of 3:1 [4]. The main symptom reported by the patients is burning, usually bilateral but sometimes also unilateral, following the distributions of one or more branches of the trigeminal. Despite any intraoral site being involved, the tongue appears to be the most frequently involved site by burning symptomatology [1,3].

Oral burning is generally associated with several oral symptoms such as subjective xerostomia, dysgeusia, a bitter/metallic taste, subjective sialorrhea, an intraoral foreign body sensation, subjective change in color or tongue morphology, tingling, itching, and globus; the early onset of the disease is characterized by a less severe symptomatology in the morning, getting worse during the day, although generally alleviated by eating and drinking [5].

This complex and different symptomatology among patients may complicate and delay the diagnosis across clinicians that generally confound the additional symptoms with others systemic diseases that may present similar abnormal oral sensations such as Sjogren syndrome [6], late-onset symptoms of COVID-19 [7] or gastroesophageal reflux disease [8]. The onset of BMS is generally spontaneous, but some patients reported antecedent dental procedures, the initiation of medications, or stressful life events are triggers of the disease [3,5].

The majority of BMS patients showed unexplained extraoral somatic symptoms such as ophthalmodynia, tinnitus, dizziness, abdominal pain or others pain conditions such as fibromyalgia, chronic fatigue syndrome, vulvodynia, low back pain and visceral pain. [9,10].

The etiopathogenesis is still debated but it is probably multifactorial in which peripheral small fiber neuropathy that involve A-delta and C-unmyelinated fibers of the trigeminal nerve, dysfunction in the brain network and psychological factors, play a role in central sensitization of the pain [3]. Recent studies on the pathophysiology of BMS have shown a decrease in peripheral nerve number and function with an overexpression of pronociceptive ion channels (transient receptor potential vanilloid 1; TRPV1) and purinergic receptors (P2 × 3) [11]. Moreover, functional Magnetic Resonance Imaging (fMRI) studies have showed several alterations in the structure and in the connections of pain matrix of the brain [12]. Indeed, significant differences have been found in the activation patterns of the brain in the BMS patients in comparison with the control group mainly in the anterior cingulate gyrus, bilateral thalamus, left lingual gyrus, bilateral precuneus, right middle frontal gyrus, right pre-central gyrus, and right inferior semilunar lobule of the cerebellum [13], a decrease in gray matter volume in medial prefrontal cortex [14] with an altered functional connectivity between the bilateral medial prefrontal cortex and amygdala and an hypoactive thalamus [15].

A recent publication points to the idea that enhanced pain perception in BMS could be linked to a higher frequency of white matter hyperintensities (WMHs) in the brain mainly in the frontal, parieto-occipital and temporal areas contributing to a premature aging of the brain in BMS patients. WMHs are considered an early marker of brain frailty and considered a consequence of cerebral small vessel arteriolosclerosis and contributing to vascular dementia but also associated with the pathogenesis of Alzheimer’s disease because ischemic insults lead to an increased expression of amyloid precursor proteins. Therefore, in this context BMS may represent a risk factor of neurodegenerative diseases in the years [16,17]. This hypothesis has been further supported by the study of Canfora et al. in which the authors reported that BMS patients, at time of diagnosis, already exhibit a preclinical transitional state of cognitive impairment called “burning fog” with subjective concentration difficulties, forgetfulness, mental confusion, and inability to multitask, with a decrease in global cognitive functions, elective attention, sustained attention, cognitive flexibility, working memory, and executive functions, outlined by a complete neurocognitive assessment [18].

The role of mood disorders in the development of BMS is not completely clear because there is still a controversy as to whether psychogenic factors are primary or secondary events in BMS patients, but it is certain that they are aggravating factors of BMS and target of the treatment. Recent evidence suggested that anxiety, depression, and sleep disturbances are the most common psychological comorbidities associated with BMS [19,20]. The overlap of BMS with mood disorders inevitably increases the burden of diseases causing a poor quality of life of patients affected. [19,21]

Moreover, BMS patients generally suffered from more comorbidities and consumed more medications than controls with a consequently poor general health status [22]. Hypertension, hypercholesterolemia, hyperhomocysteinemia, hypothyroidism, and gastroesophageal reflux in addition with mood disorders are the most common medical comorbidities associated with BMS [16,18,23].

The diagnosis of primary BMS is a clinical challenge and can only be established by exclusion of other systemic or local disorders that may cause oral cavity pain; therefore, the clinicians, after a careful clinical examination of the oral cavity, should collect a comprehensive medical history, the current medications, and psychiatric diseases history [23]. Moreover, it is essential to analyze the characteristics of pain (onset, duration, location, exacerbating/ameliorating factors) also with appropriate qualitative and quantitative pain tools. In addition, it is fundamental to evaluate the psychological and sleep profile which could also amplify the pain perception [24]. A correct BMS diagnosis should include the metabolic profile evaluation through hematological tests, to rule out the nutritional, hormonal, autoimmune [25] and thrombophilia conditions [16,18]. Finally, an MRI of the brain should be considered as an adjunctive tool in the assessment of BMS patients in order to identify WMHs and to evaluate the cardiovascular risk of the patient [16,18].

The complexity of the BMS patients requires a multidisciplinary approach with a strong collaborative relationship between orofacial pain specialist, physician, psychiatrist, and neurologist. A good alliance between the specialist and patient, through the exchange of information about the disease and the reassurance about its benignity, is essential and it should be considered a therapeutic intervention [25].

Currently, the pharmacological intervention of BMS follows the patient-centered model in which the cornerstone is to firstly treat the patient rather than the disease [26]; therefore, the clinicians should choose specific drugs useful to alleviate the pain and to improve mood and sleep quality in order to improve the quality of life of any patients affected. Despite there being no evidence of the efficacy of specific medications or agreement between the authors, various neuropathic medications such as Clonazepam, Gabapentin, Tricyclics, Cannabinoids have been proposed for the treatment of BMS.

Recently, Vortioxetine, a multimodal antidepressant, has demonstrated its efficacy in the pain relief, anxiety, depression, and sleep disturbance in BMS. In addition, this drug may improve cognitive impairment and preserve the brain from a future damage. No multi-drug interactions or side effects, such as QTc prolongation, sexual dysfunction or weight gain has generally reported with the use of Vortioxetine; for its good tolerability and efficacy this drug may be considered a new frontier in the management of this disease but also in other chronic pain conditions [18,27].

Moreover, it seems to be essential to monitor and to treat modifiable cardiovascular risk factors, promoting correct lifestyle behaviors in order to prevent and/or reverse the WMHs and consequently delay neurodegenerative diseases [16].

BMS remains a poorly understood disease across healthcare providers with a long-lasting diagnostic delay that compromise the efficacy of the treatment for this reason it seems essential to improve the knowledge about this disease in every specialty of medicine through educational intervention in order to ensure an early diagnosis, improving the prognosis and treatment of patients suffering from BMS [28].
